# Masking Agents Evaluation for Lead Determination by Flow Injection-Hydride Generation-Atomic Fluorescence Spectrometry Technique: Effect of KI, L-Cysteine, and 1,10-Phenanthroline

**DOI:** 10.1155/2016/3095120

**Published:** 2016-04-11

**Authors:** Blanca G. Beltrán, Luz O. Leal, Laura Ferrer, Víctor Cerdà

**Affiliations:** ^1^Environment and Energy Department, Advanced Materials Research Center (CIMAV) S. C., Miguel de Cervantes 120, 31136 Chihuahua, CHIH, Mexico; ^2^Department of Chemistry, University of the Balearic Islands, Cra. Valldemossa km 7.5, 07122 Palma, Spain

## Abstract

Hydride generation (HG) of lead technique presents interferences from foreign ions of complex matrix samples. In order to minimize these interferences, the effect of masking agents such as KI, L-cysteine, and 1,10-phenanthroline was studied in the absence and in the presence of selected interfering species (As, Cr, Cu, and Fe). Different modes of addition of masking agents were accomplished, that is, to either sample or KBH_4_ reducing solution. The lead determinations were performed using a flow injection analysis (FIA) system coupled to HG and atomic fluorescence spectrometry (AFS). The linearity of calibration curves (1–10 *μ*g Pb L^−1^) was not affected by the addition of the masking agents. The use of KI in the reducing solution diminished interferences from concentrations of As and Cu, while 1,10-phenanthroline showed a positive effect on the interference by As. Moreover, Cr and Cu appeared to be the most serious interfering ions for plumbane (PbH_4_), because they drastically reduced the analytical signal of lead. Fe did not present any interference under the employed experimental conditions, even at high levels. The accuracy was established through the analysis of certified reference material (i.e., BCR-610, groundwater) using KI as masking agent. The detection limit reached by FIA-HG-AFS proposed methodology was 0.03 *μ*g Pb L^−1^.

## 1. Introduction

The determination of ultra-traces of lead in environmental samples plays an important role in the pollution monitoring [1]. Among several techniques used to measure lead concentrations, atomic fluorescence spectrometry (AFS) has been widely used as a detection technique for the analysis of different kinds of biological and environmental samples [2, 3]. However, in the case of the analytical determination of trace amounts of lead, the direct analysis is inadequate, and it is necessary to improve the sensitivity of AFS by using hydride generation (HG) of its volatile species (PbH_4_) [4]. The hyphenation of HG for lead separation from the matrix sample [5, 6] with AFS detection exploiting a flow-based technique offers sensitive detection and fast operation [7].

Volatile hydrides of As, Sn, Sb, Cd, Se, Hg, and Pb [8] are usually generated using tetrahydroborate (THB) in acid medium (HCl, HNO_3_), which is capable of producing borane complexes [9]. Furthermore, volatile species arising out of the transition metals such as Fe, Cu, and Cr also can be formed. These vapour species are highly unstable and can readily form colloids or macro precipitates [10, 11].

In the case of Pb, the generation of plumbane is achieved by using THB in combination with potassium ferricyanide K_3_[Fe(CN)_6_], which is one of the most used additives improving the generation of plumbane [4, 12]. The reaction of potassium ferricyanide with THB forms hydroboron intermediates which can react efficiently with Pb giving strong enhancement of plumbane generation reaction [13]. The generation of lead hydride is difficult and different from other hydride-forming elements because it is highly susceptible to suffering interferences, which can be produced by foreign ions present in sample matrices. PbH_4_ can only be achieved in the presence of oxidizing and chelating or masking agents (MA) [14]. These additives are chemical substances which are added to the reaction system in order to improve both the efficiency and the selectivity of plumbane generation [15].

Serious interferences derive from noble metals, transition metals (e.g., Cu, Fe, and Cr), and hydride-forming elements (As), especially when they are present in great excess over the analyte [16].

The interference effects are acutely different and unpredictable, being dependent on the specific analyte-interfering couple, experimental conditions (acid and THB concentration, type of acid), the type and design of the apparatus (continuous flow, flow injection, and batch), detectors employed, and performances of the reactor and gas-liquid separation units of HG system [11, 15–17]. Different interference mechanisms have been set by categories as follows: analyte-foreign ion; analyte-product interference; hydride-foreign ion interference; hydride-product interference; THB depletion interference. Previous works have reported that most of these mechanisms are effective in explaining the interferences in HG [18, 19].

The interferences can occur before or during formation of the hydride, in the separation of the volatile species from the liquid phase, and also during atomization [15]. Liquid-phase interferences are strictly related to the mechanism of HG with borane complexes; however these mechanisms are not clear enough [6]. Furthermore, gas-phases interferences can occur due to the formation of other hydrides, volatiles species of transition elements and aerosol transported with carrier gas [20].

The use of MA in HG technique for trace elements determination is one of the most important methods for the control of liquid-phase interferences generated by transition metals and hydride-forming elements, reducing interferences and maintaining the analytical sensitivity [21]. The mode of addition of MA has a great effect in the control of interfering processes, which could explain the discrepancies reported in the literature [22]. MA can be added on-line or as batch to reducing solution or to sample before or after HG.

However, in the literature there have been scarce reported works about the lead determination by HG-AFS in environmental samples using MA in order to reduce or avoid interferences. Since details of operating conditions of HG reaction (e.g., acidification, reagent concentrations, order to add the MA, and flow rates) are missing in the bibliography, the results about chemical interferences in plumbane generation are difficult to compare [23].

Several reagents such as L-cysteine, 1,10-phenanthroline, and potassium iodide (KI) were used as MA to reduce chemical interferences caused by foreign ions. L-Cysteine with THB played a role in both signal enhancement and interference control of germane-HG [24]. In addition, L-cysteine did not affect the hydride generation of Sn utilizing a reaction medium at lower acidities [25]. 1,10-Phenanthroline has been used as additive for the control of interferences in the determination of arsenic and selenium and it improved tolerance limits of Ni(II) and Cu(II) [26, 27]. Other works have described batch procedures by AFS for Pb(II) determination in vegetal and geological samples [28, 29]. Besides, interferences were avoided by the addition of phenanthroline-thiocyanate and oxalic acid as MA [14]. Some works have reported the addition of the KI to reducing solution of THB, which has been effective in the control of transition metal interferences [30, 31]. This has been attributed to the catalytic effect of halide ions on the formation of hydrides. Furthermore, the addition of KI to THB solution in combination with thiourea as MA resulted in a suitable MA for selenium and tellurium determination [31].

To the best of our knowledge, at present, the use of KI, L-cysteine, and 1,10-phenanthroline as MA in plumbane generation has not been evaluated comparatively in order to abate the liquid-phase interferences caused by foreign ions. Thus, the main objective of this work is the evaluation of the above-mentioned MA to solve the negative impact of As, Cu, Fe, and Cr in the analytical signal of Pb by FIA-HG-AFS technique.

## 2. Experimental Section

### 2.1. Reagents

All chemicals used were of analytical reagent grade. For preparation of all solutions, Millipore-purified water was used. Glassware needed for lead determination was soaked in 10% (v/v) HNO_3_ (65%) and rinsed with Millipore water.

Series of Pb, As, Cu, Cr, and Fe standard solutions were prepared by gradually diluting 1000 mg L^−1^ standard stock solutions from CENAM (National Metrology Center, Mexico) in 50 mL of 1.5% (v/v) HCl (36–38%) solution.

A 1% KBH_4_ (98%, Sigma-Aldrich) and 1.5% (m/v) K_3_[Fe(CN)_6_] (99.5%) solution as reducing solution were daily prepared with a 0.2% KOH (87%) solution.

L-Cysteine (97%), 1,10-phenanthroline (99%), and KI (99.6%) were used to prepare MA solutions. The L-cysteine and 1,10-phenanthroline solutions were added to standards solutions and were acquired from Sigma-Aldrich. KI was added to reducing solution KBH_4_.

Nitric and hydrochloric acids, potassium ferricyanide, potassium hydroxide, and potassium iodide were purchased from J.T.Baker, USA.

### 2.2. AFS Set-Up and Manifold

Measurements were accomplished by means of an atomic fluorescence spectrometer AF-640 (Rayleigh Analytical Instrument Corp., Beijing, China). The instrument was equipped with a gas-liquid separator (GLS), which allows the separation of the PbH_4_ of the aqueous phase of the sample. A super-cathode lamp was used as radiation source (*λ* = 283 nm). The manifold consists of a peristaltic pump with two sampling tubes (1.59 mm id): one of them for reducing solution and the other one for sample/carrier solution. The manifold comprises a holding coil (HC), a mixed module (MM), and a reaction coil (RC). An argon gas input was set after RC. In Table 1 are summarized the operating parameters of the AF-640.

Data collection and processing as well as the instrumental control were carried out with the manufacturer software AF-640 1.3.

### 2.3. Working Conditions and Procedure

Since the efficiency of HG reaction, that is, the degree of the release of lead hydride in a reaction/separation cell and the transport to the detection system, depends critically on the adjustment of chemical and flow parameters, these conditions were optimized in a previous work [32]. The reagent concentrations were employed as follows: potassium ferricyanide solution was set in 1.5% (m/v), potassium borohydride solution was set in 1% (m/v), and hydrochloric acid solution was set in 1.5%.

**Figure 1:**
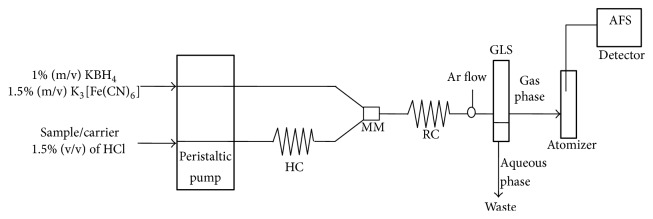
Schematic representation of flow-based hydride generation-atomic fluorescence spectrometry system, FIA-HG-AFS. HC: holding coil; MM: mixed module; RC: reaction coil; GLS: gas-liquid separator.

A scheme of FIA-HG-AFS system for Pb determination is showed in Figure 1. First, the sample is sucked into the HC, but it does not enter in the mixed module (MM); therefore the vapour has not been generated yet. At the same time the solutions of KBH_4_ and K_3_[Fe(CN)_6_] are also impelled by the peristaltic pump and go inside to the RC. The peristaltic pump stops running; the sample collection is complete. The sample/carrier tube is manually transferred to the carrier. The carrier (1.5% m/v HCl) drives to the sample through a MM and then the sample is introduced into RC, where the hydride generation is performed. The volatile lead hydride and the hydrogen gas produced by the reaction are carried by the argon gas into the quartz furnace atomizer to form the Ar-H_2_ flame.

### 2.4. Optimization of the Masking Agent Concentrations

The effect of MA on the lead analytical signal by FIA-HG-AFS was first tested in the absence of foreign ions, on the slope of calibration curves in a concentration range of 0–10 *μ*g L^−1^ of Pb. In addition, different concentrations of MA were utilized in each curve, to prove if there was loss of sensitivity in the method, namely, if the presence of MA caused them some kind of signal suppression. With these results, an adequate concentration of each MA was selected, which was utilized in the posterior experiments.

### 2.5. Effect of Masking Agents on the Plumbane Generation in Presence of Foreign Ions

The effect of MA on the analytical signal of lead was tested in presence of different concentrations of interferents, namely, As, Cu, Cr, and Fe, using a lead standard solution of 10 *μ*g L^−1^ (Table 2). The concentration levels of each one of the foreign ions were set taking into account their concentration in environmental samples. Recoveries were evaluated in order to find the maximum tolerated concentration of these foreign ions in the PbH_4_ generation. The criterion to consider that an element does not interfere was that the variation of the analytical signal is lesser than ±10%. Finally, the interfering tolerated concentration in presence of MA was established.

### 2.6. Application of Masking Agents in BCR-610 Certified Reference Material

The use of MA in the determination of lead by HG-AFS was validated by the analysis of a certified reference material BCR-610 (groundwater) of the Community Bureau of Reference. The addition of KI to THB solution was carried out with the purpose of masking interferences, allowing the achievement of reaction conditions and obtaining results in agreement with the certified reference values.

## 3. Results and Discussion

### 3.1. Optimization of the Masking Agents Concentrations

**Figure 2:**
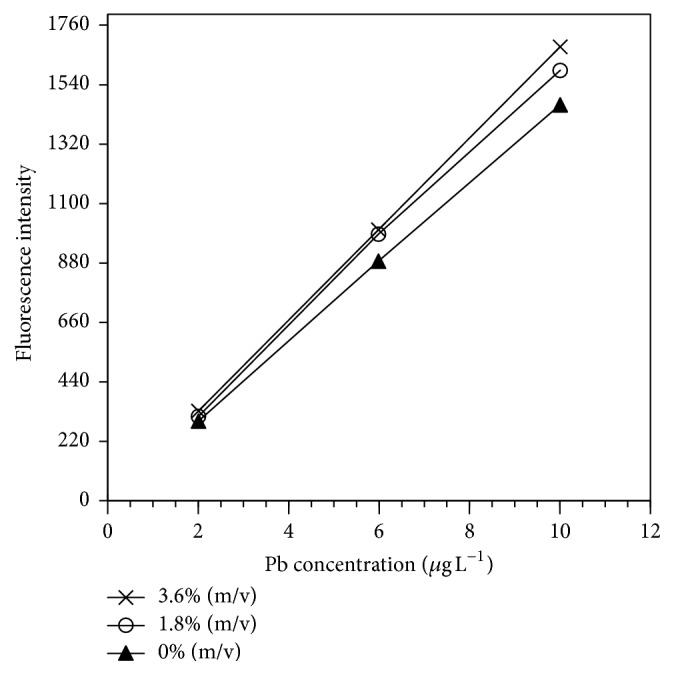
Calibration curves for lead obtained in the presence of different concentration of KI added to the reducing solution. Reaction conditions: 1% (m/v) KBH_4_, 1.5% (v/v) HCl, and 1.5% (m/v) K_3_[Fe(CN)_6_].

In the absence of interfering species, KI produced a slight enhancement in the sensitivity, which is increasing as the KI concentration increases (Figure 2), due to the catalytic role of iodide [33], which is consistent with a previous study for hydrogen telluride generation [31]. Thus, a concentration of 1.8% (m/v) KI was selected for subsequent experiments with foreign ions, since at higher concentrations a major increase in the analytical signal was not observed.

**Figure 3:**
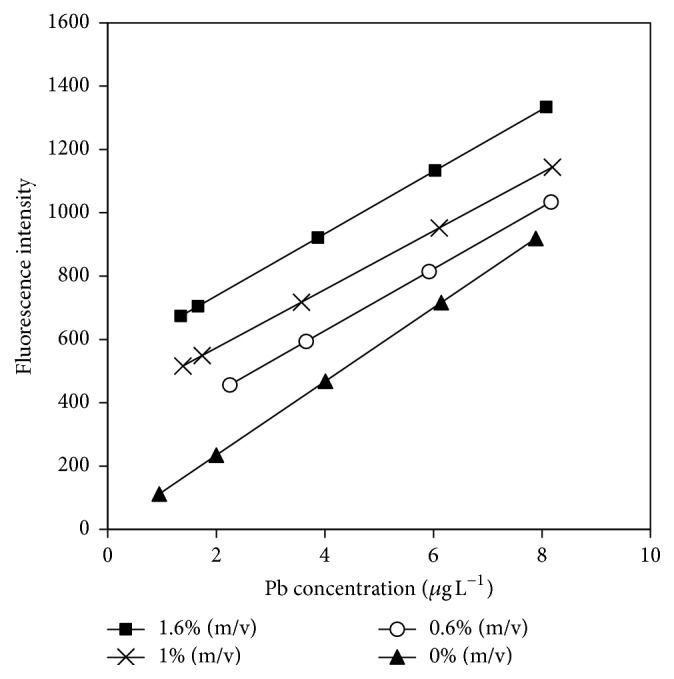
Calibration curves for lead obtained in the presence of different concentration of L-cysteine. Reaction conditions: 1% (m/v) KBH_4_, 1.5% (v/v) HCl, and 1.5% (m/v) K_3_[Fe(CN)_6_].

Results showed that as L-cysteine concentration increased, the sensitivity decreased, and the values of the blanks were higher. Therefore, seeking a compromise between sensitivity and matrix interference, the selected concentration was 0.6% (m/v) (Figure 3).

**Figure 4:**
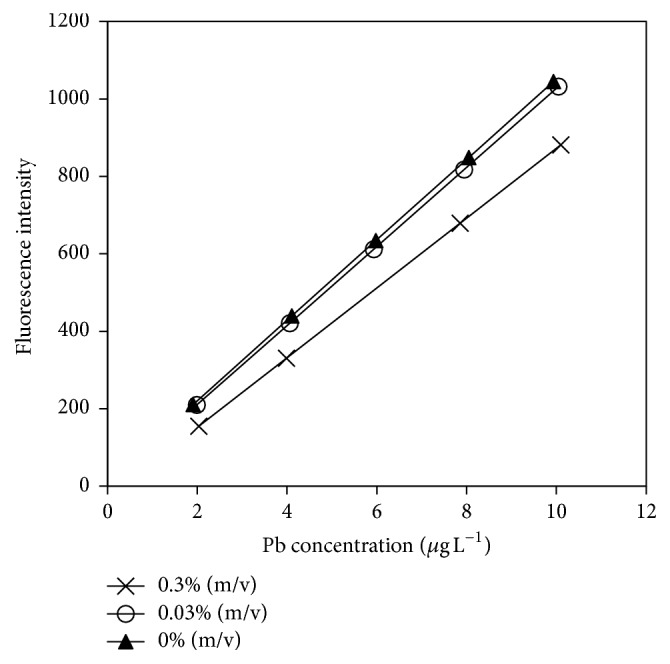
Calibration curves for lead obtained in the presence of different concentration of 1,10-phenanthroline. Reaction conditions: 1% (m/v) KBH_4_, 1.5% (v/v) HCl, and 1.5% (m/v) K_3_[Fe(CN)_6_].

Meanwhile, 1,10-phenanthroline was tested at two different concentrations. A concentration of 0.03% (m/v) 1,10-phenanthroline was selected, considering that the corresponding slope of the calibration curve remained almost parallel to that corresponding to the calibration curve without 1,10-phenanthroline (Figure 4).

In summary, the concentrations of each MA used in the following tests to evaluate its effect on the foreign ions in the generation of plumbane were 1.8% (m/v) KI, 0.6% (m/v) L-cysteine, and 0.03% (m/v) 1,10-phenanthroline.

### 3.2. Effect of Masking Agents on Foreign Ions in the Plumbane Generation

**Figure 5:**
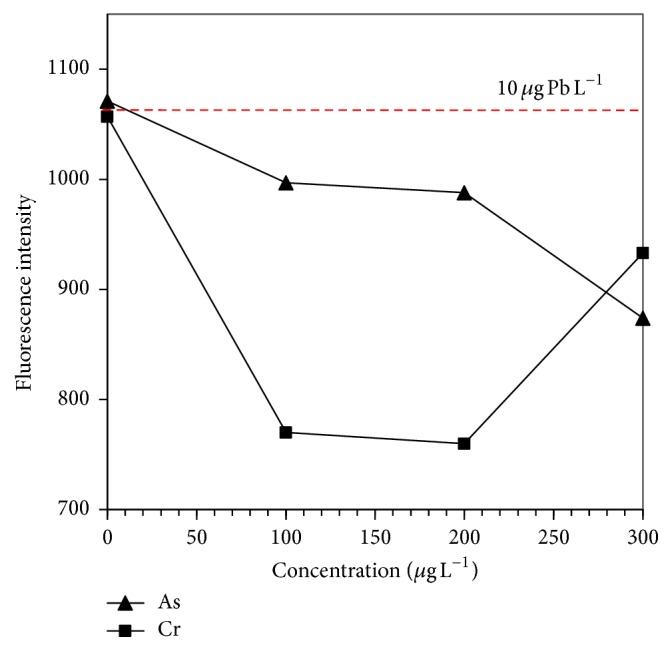
Effect of As and Cr concentrations on the fluorescence intensity of Pb. Peak height measurements for 10 *μ*g Pb L^−1^.

**Figure 6:**
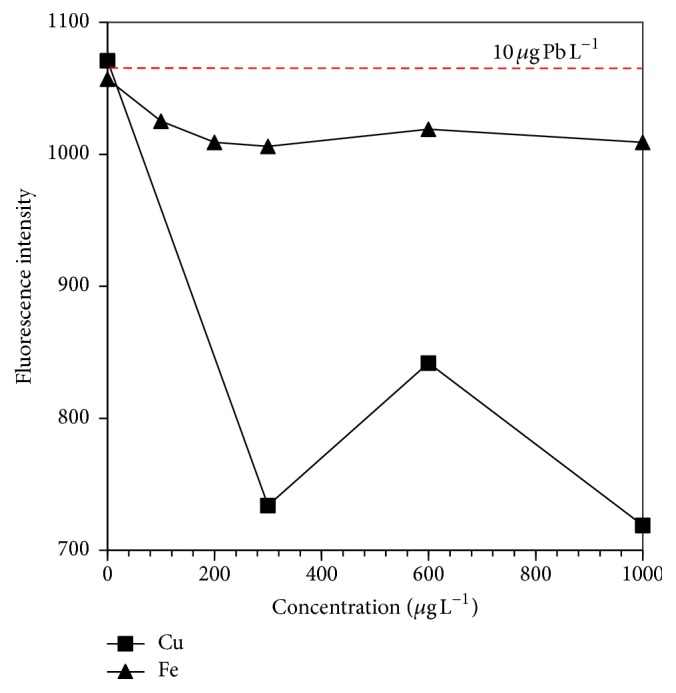
Effect of Cu and Fe concentrations on the fluorescence intensity of Pb. Peak height measurements for 10 *μ*g Pb L^−1^.

The depressive effects of As^+5^, Cr^+6^, Cu^+2^, and Fe^+3^ on the analytical signal (i.e., fluorescence intensity (FI)) obtained in the lead hydride generation of 10 *μ*g Pb L^−1^ are shown in Figures 5 and 6. It is presumed that the mechanism of interference in the liquid phase is based on the interaction between tetrahydroborate and the metal ions [8]. It is important to mention that the transition metals such as Fe, Cu, and Cr can also form volatile species by HG reactions, and gas-phase interferences could occur.

It should be pointed out that Fe did not cause a severe interference on the plumbane generation until a concentration tested of 1000 *μ*g Fe L^−1^, which was even higher than that found in drinking and river water. The recoveries were maintained above 95%. Then, the addition of any MA was not necessary.

The mechanism of the MA in HG is explained based on the analyte-MA and interferent-MA (additive) interactions [6]. Thus, different modes of addition of MA were accomplished, obtaining the best results when KI was added to the reducing solution KBH_4_ and L-cysteine and 1,10-phenanthroline were added to the sample.

#### 3.2.1. Arsenic

The reaction system for the plumbane generation is formed by Pb-THB-K_3_[Fe(CN)_6_]-HCl interactions. The addition of As affected the generation of PbH_4_, since the recoveries of Pb decreased until 82% in presence of 300 *μ*g As L^−1^ (Table 3). It is probably due to THB depletion interference, in which this reagent is preferentially consumed by the interferent. Some analytical substrates such as As are more active than others and react with almost all the hydroboron species [6]. Thus, the rate of reaction of Pb with THB plays an important role on the generation of plumbane [34] and on the interference mechanism.

So, an inadequate concentration of THB could cause an inefficient reaction between THB and K_3_[Fe(CN)_6_] and at the same time diminishes the borane complex intermediates, which are highly effective in the generation of plumbane [13].

However, it was found that it is possible to restore the lead analytical signal by the addition of KI to the reducing solution, obtaining 92% of recovery at the same concentration of As. Thus, a concentration of 300 *μ*g As L^−1^ was set as the tolerance limit concentration, utilizing KI.

L-Cysteine failed to have a notorious masking effect, because the analytical signal of Pb was decreased in the presence of each one of the As concentrations tested in the experiments. L-Cysteine could have a masking effect by two proposed mechanisms. First, L-cysteine-THB complexes can be formed with L-cysteine and KBH_4_ reducing solution, which could be precursors to improvement of the HG efficiency of the target analyte [6]. On the other hand, L-cysteine-analyte complexes also can be formed, which are promoted at lower acidities, whereas L-cysteine-THB complexes are formed at higher acidities [25]. Following this approach and considering the low acidities conditions for plumbane generation, L-cysteine-THB could not be formed. On the other hand, the formation of the L-cysteine-analyte complexes was not favoured, even with favourable acidic conditions, since a masking effect for arsenic interference was not observed.

Otherwise, 1,10-phenanthroline as MA had the same effect of KI, improving the Pb analytical signal until 93% at 300 *μ*g L^−1^ As concentration. Previous work has reported the use of 1,10-phenanthroline as MA, which can react with K_3_[Fe(CN)_6_], maintaining the reaction conditions for plumbane generation [14].

#### 3.2.2. Copper

Copper is recognized as a species able to produce a serious interference in the chemical generation of volatile species of hydride-forming elements [15]. In addition, Cu can also be converted into a volatile species and a noticeable aspect is that in its determination by HG it is markedly free from interference effects [35].

Low Pb recoveries were obtained as a result of the presence of different Cu concentrations tested (Figure 6). This fact could be explained based on the reagent- (THB-) interferent interaction; that is to say, the Cu concentration increases the rate of tetrahydroborate hydrolysis causing incomplete formation of the Pb hydride [30]. The presence of Cu can lead to the formation of THB-interferent complexes, which can involve toward the formation of reaction products of the interferent [15]. These products could cause an interference of the type hydride-product interference, in which the plumbane could be captured by foreign species (products of the interferent) [36].

The use of KI allowed improving the Pb maximum tolerance limit until 600 *μ*g Cu L^−1^ (Table 3). It has been reported in previous works that KI can mask the Cu ion interference due to the formation and precipitation of CuI or due to the possible presence of other species in solution such as Cu in ground state, I_2_, or a combination of them [21, 30, 37]. No precipitate in this work was observed. An explanation of this could be the chemical conditions (sample acidity, KI and Cu^+2^ concentrations) used in this work. In a previous study for selenium determination [21], the sample acidity was 5–16 times higher than that presented in this work and KI concentration was 5 times higher, whereas the Cu^+2^ concentrations tested were about 33–1000 times higher than those assayed in this work.

Thus, based on the literature cited above, the additive-interferent interaction (i.e., KI and Cu) could cause the formation of complex and redox reactions [15]. A concentration of 600 *μ*g Cu L^−1^ was set as the tolerance limit concentration utilizing KI as MA.

L-Cysteine did not have a MA effect on the Cu ions at the assayed concentrations. It should be noted that both MA and interferent produced a perturbation on the plumbane reaction system. The experiment results were displayed in Figure 3 and Table 3.

The 1,10-phenanthroline increased the analytical signal of lead, obtaining recoveries outside of the tolerated limit of interferences set in ±10%. Reagent-additive interaction can modify the mechanism and the kinetics of hydrolysis of borane reagent allowing the formation of additive-borane complexes [15]. However, an interaction between 1,10-phenanthroline and K_3_[Fe(CN)_6_] can also be considered [14].

#### 3.2.3. Chromium

The liquid-phase interference mechanism considered for transitions metals such as Cr is one in which reactions between THB, analyte, and interfering ion conduced to the formation of metal colloids/metal borides. These chemical species have interaction with the analyte hydride causing interferences [6].

In this study, Cr appeared to be one of the most serious interfering ions for plumbane generation, because its presence causes lead recoveries between 73 and 88% (Table 3 and Figure 5). There is no improvement on the analytical signal of lead, since all MA had almost the same behaviour as the standard solutions in which MA were not used. Thus, it could be inferred that none of the MA were effective on different concentrations tested of Cr (Table 3). However, KI showed a slight improvement in the solution that contained 300 *μ*g L^−1^ of Cr (90% of lead recovery).

### 3.3. Validation with BCR-610 Certified Reference Material Using KI as Masking Agent

The BCR-610 certified reference material was used to validate the use of MA in lead determination exploiting FIA-HG-AFS system. A “*t*-” test was applied in order to establish the statistical significance of results.

First, a direct analysis of BCR-610, that is, without MA, was performed. The results showed significant differences, which were found at confidence level of 95%, between the certified value of 7.78 ± 0.13 *μ*g Pb L^−1^ and the obtained value of 6.58 ± 0.07 *μ*g Pb L^−1^. The low recovery can be due to the contents of As and Cu in BCR-610, 10.8 and 45.7 *μ*g L^−1^, respectively. In addition, double peaks were obtained in the lead measurement, which is typical of interference phenomena and it is due to the slower rate of formation of one of the interfering species that is formed with a slower rate in respect to the analyte hydride [38]. However, with the use of KI as MA only one peak was observed and a value of 7.75 ± 0.08 *μ*g Pb L^−1^ was achieved. Thus, this result was in agreement with the certified value and also indicated the positive effect of KI in the control of interference generated by relatively high concentration of As and Cu.

## 4. Conclusions

The evaluation of MA and their effects in minimizing interferences in the plumbane generation, exploiting AFS as detector, was carried out. Excepting Fe, which did not cause interference on the plumbane generation even at a high concentration, the rest of the foreign ions tested, that is, As, Cu, and Cr, promoted interferences in absence of MA. THB depletion interference was caused by the presence of As. Transition metals such as Cu and Cr were the most serious interfering ions because they drastically reduced the analytical signal of lead, due probably to two types of interferences: hydride-product interference and hydride-ion interference. The sensitivity of the method was not affected with the use of KI and 1,10-phenanthroline, since no changes occurred in the slope of the calibration curves for both MA. On the other hand, L-cysteine produced high values of the blanks and a decrease in sensitivity. In addition, it had no effect at any concentration tested of the foreign ions. KI added to the reducing solution (KBH_4_) diminishes As and Cu interferences. 1,10-Phenanthroline as MA had a slight positive effect for As, but it did not have effect for Cu. Despite the fact that the Pb analytical signal was increased in presence of Cu with 1,10-phenanthroline, the recoveries were out of the tolerated limit range of interferences, probably because of an interaction between 1,10-phenanthroline and THB or its interaction with K_3_[Fe(CN)_6_]. The presence of MA did not have effect in the interference caused by Cr. The determination of lead at trace levels in the BCR-610 (groundwater) certified reference material was satisfactory using KI as MA.

## Figures and Tables

**Table 1:** Operation parameters of AFS instrument and FIA system.

AFS parameters		Chemical conditions	
Argon flow rate (mL min^−1^)	400	KBH_4_	1% (m/v)
Delay time (s)	4	K_3_[Fe(CN)_6_]	1.5% (m/v)
Reading time (s)	22	Carrier HCl	1.5% (v/v)
Load sample time (s)	8	HCl medium for sample and standard solutions	1.5% (v/v)
Sample volume (mL)	1.3		
Sampling loading flow rate (mL min^−1^)	8		

**Table 2:** Assays carried out for foreign ions.

Foreign ion	Levels of concentration (*µ*g L^−1^)	Masking agent (MA)
Arsenic	0, 100, 200, 300	Without MA KI L-Cysteine 1,10-Phenanthroline

Copper	0, 300, 600, 1000	Without MA KI L-Cysteine 1,10-Phenanthroline

Chromium	0, 100, 200, 300	Without MA KI L-Cysteine 1,10-Phenanthroline

Iron	0, 100, 200, 300, 600, 1000	Without MA

**Table 3:** Results of the effect of foreign ions and masking agents on the analytical signal of 10 *µ*g Pb L^−1^.

Foreign ion concentration (*µ*g L^−1^)	^a^FI without masking agent	Recovery (%)	FI with KI	Recovery (%)	FI with L-cysteine	Recovery (%)	FI with 1,10-phenanthroline	Recovery (%)
^b^Arsenic								
0	1071 ± 4		1036 ± 2		1026 ± 2		1089 ± 12	
100	997 ± 3	93	1043 ± 6	101	995 ± 5	97	1097 ± 7	101
200	988 ± 7	92	968 ± 5	93	844 ± 9	82	1016 ± 10	93
300	874 ± 0.2	82	887 ± 3	92	681 ± 4	66	1009 ± 8	93

^b^Copper								
0	1071 ± 9		1036 ± 11		1026 ± 2		1071 ± 9	
300	734 ± 5	69	935 ± 7	90	631 ± 5	61	1442 ± 17	135
600	842 ± 7	79	842 ± 7	90	627 ± 25	61	1442 ± 25	135
1000	719 ± 4	67	719 ± 4	85	477 ± 2	46	1395 ± 17	130

^b^Chromium								
0	1057 ± 4		1036 ± 11		950 ± 4		1071 ± 9	
100	770 ± 9	73	755 ± 9	73	763 ± 3	80	768 ± 7	72
200	760 ± 12	72	762 ± 2	73	747 ± 12	79	768 ± 16	72
300	933 ± 13	88	932 ± 1	90	675 ± 5	71	768 ± 20	72

^b^Iron								
0	1057 ± 4							
100	1025 ± 16	96						
200	1009 ± 13	95						
300	1006 ± 8	95						
600	1019 ± 17	96						
1000	1009 ± 7	95						

^a^FI: fluorescence intensity.

^b^Measurements reported as the mean ± SD (*n* = 3).
